# Patient Burden in Dystonia Diagnosis and Botulinum Toxin Treatment: A Nationwide Survey in Turkey

**DOI:** 10.1002/brb3.70325

**Published:** 2025-02-28

**Authors:** Rezzak Yilmaz, Nevra Öksüz, Mustafa Ceylan, Bedia Samanci, Ahmet Acarer, Nazlı Durmaz Çelik, Hacer Erdem Tilki, Serhat Özkan, Haşmet Hanağası, Okan Dogu, M. Cenk Akbostancı

**Affiliations:** ^1^ Department of Neurology Ankara University School of Medicine Ankara Turkey; ^2^ Brain Research Center Ankara University Ankara Turkey; ^3^ Department of Neurology Mersin University School of Medicine Mersin Turkey; ^4^ Department of Neurology Atatürk University School of Medicine Erzurum Turkey; ^5^ Behavioral Neurology and Movement Disorders Unit, Department of Neurology Istanbul Faculty of Medicine, Istanbul University Istanbul Turkey; ^6^ Department of Neurology Ege University School of Medicine Izmir Turkey; ^7^ Department of Neurology Osmangazi University School of Medicine Eskisehir Turkey; ^8^ Departments of Neurology and Clinical Neurophysiology Ondokuz Mayıs University School of Medicine Samsun Turkey

**Keywords:** alternative treatment, blepharospasm, burden, cervical dystonia, dystonia, hemifacial spasm, oromandibular dystonia, task‐specific dystonia

## Abstract

**Background:**

Understanding real‐world experiences is crucial in determining the potential gaps in patient‐centered healthcare in dystonia. We explored the challenges of people with dystonia (PwD) at the stages of diagnosis and botulinum neurotoxin (BoNT) treatment.

**Methods:**

A multicenter survey was conducted face‐to‐face across seven university hospitals in Turkey. PwD receiving BoNT treatment for at least 6 months were invited. Data on demographics, diagnostic journeys, and treatment experiences were collected and analyzed using descriptive statistics and regression models.

**Results:**

A total of 789 PwD participated, reporting significant burdens during both diagnostic and treatment stages. Diagnosis was delayed by approximately 1 year, with 15% receiving incorrect initial diagnoses. Additionally, 30.7% of PwD traveled to another city, and 42.6% applied to private clinics for diagnosis, leading to a substantial logistic and financial burden. The treatment stage revealed that a quarter of PwD had to travel significant distances every 3 months, or applied to a private clinic to receive BoNT injections, creating considerable cost and effort. In addition, PwD with oromandibular dystonia were three times and task‐specific dystonia were around nine times less likely to be satisfied with BoNT treatment compared to facial dystonia. Alternative treatment was reported in 11%, with no substantial benefit.

**Conclusions:**

The findings highlight critical unmet needs in the diagnostic and treatment processes for PwD. These include improvement in diagnostic accuracy, reduced travel and financial burdens, and enhanced treatment satisfaction. An action plan emphasizing resource utilization policies and educational activities for healthcare providers is essential to address these challenges.

## Graphical abstract

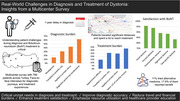

## 1 Introduction

In 2013, an international consensus committee published a consensus update defining dystonia as “Sustained or intermittent muscle contractions causing abnormal, often repetitive, movements, postures or both” (Albanese et al. 2013). The wording of this definition implies the difficulties in characterizing the phenomenology of dystonia. It includes the conjunction “or” twice, indicating differences in the duration and the type of the movement, and the adverb “often,” indicating that it may sometimes present otherwise. Moreover, apart from various temporal patterns (such as persistent, diurnal, or paroxysmal), dystonia manifests in different body parts, at different ages, and be accompanied by other movement disorders (Albanese et al. 2013). These features underline the variability in presentation and suggest that recognition and diagnosis of dystonia in the clinical routine may be challenging even for movement disorder specialists, let alone the neurologists or general practitioners who probably encounter people with dystonia (PwD) first (Benson et al. 2022; Smit et al. 2021). These challenges may lead to delayed or incorrect diagnosis negatively affecting the lives of PwD.

In addition to the challenges at the diagnostic stage, access to treatment may not be easy, especially for PwD, for whom a chemodenervation using botulinum neurotoxin (BoNT) is the choice of treatment (C. Comella et al. 2021). Treatment with BoNT is invasive, requires trained personnel, and experience in assisting devices (electromyography and ultrasound). Furthermore, in almost all PwD, BoNT treatment is long term with 3–4 annual repetitions, underscoring the need for sustainable, expert‐level medical care (Tamás et al. 2020). Therefore, the treatment stage is also inherent to challenges that may have a significant negative impact on the quality of life of PwD (Smit et al. 2021; Girach et al. 2019; Drexel et al. 2020). Previous studies investigating these impacts reported that dystonia is associated with various challenges, including difficulties at work, sleep disturbances, vision problems, sexual activity issues, lack of energy, diminished self‐confidence, and symptoms of depression or anxiety (C. Comella et al. 2021; Drexel et al. 2020).

These potential challenges extend beyond the clinical field into areas of healthcare organizations, economics, and policies. To that end, coalitions or networks have been established to address the unmet needs of PwD and healthcare providers that provided invaluable insights (Smit et al. 2021; Colosimo, Bhidayasiri et al. 2019; Centen et al. 2023). However, to fully understand the hardships of PwD, more patient‐oriented data from diverse populations are needed. Thus, in this nationwide survey study, we aimed to explore patient burden in diagnosis and access to BoNT treatment in PwD.

## 2 Methods

The movement disorder centers of seven university hospitals in Turkey that serve as tertiary reference centers participated in this multicenter study. Each center represented one of the seven regions of the country. Between October 2022 and 2023, PwD receiving BoNT injections for the treatment of focal or segmental dystonia (SegD) at these centers for at least 6 months were invited to fill out a survey investigating their burden related to diagnosis and BoNT treatment. Patients with hemifacial spasm (HFS) were also included in PwD, given that they also receive BoNT treatment and may experience similar challenges. A pilot study on 20 patients was performed for cognitive testing of the survey and the final version was developed following patient feedback and expert opinions. The survey was administered through face‐to‐face interviews during their visit to outpatient clinics for BoNT treatment. The full version of the survey is given in the Supporting Information. Study data were collected and managed using REDCap electronic data capture tools hosted at the Ankara University Department of Neurology (Harris et al. 2019). Study protocol and informed consent were approved by the local ethical committee. All procedures followed the Declaration of Helsinki, and informed consent was obtained from all participants.

### 2.1 Statistical Analysis

Assuming an overall prevalence of 14.8/100,000 for all types of focal dystonia (including SegD; Epidemiological Study of Dystonia in Europe (ESDE) Collaborative Group 2000) and 11/100,000 for HFS (Nilsen et al. 2004), there are an estimated 20,640 patients in Turkey with dystonia and HFS requiring BoNT treatment. With a 99% confidence level and a 5% margin of error for such a population, the adjusted sample size calculation required the participation of at least 643 patients for a representative sampling, which was achieved (see the results). Responses to the survey were analyzed using descriptive statistics without hypotheses. Results are presented as mean (standard deviation [SD]), median (range), or percentages according to the type and distribution of the data. Explorative comparisons including contingency tables and regression models were performed between types of dystonia or location of PwD to demonstrate different patient experiences. SPSS Statistics 22.0.0 (SPSS Ltd., Chicago, IL) was used for statistical analyses.

## 3 Results

The participating centers representing seven regions of Turkey were the university hospitals of Mersin, Ankara, Erzurum, Istanbul, Izmir, Eskisehir, and Samsun, which recruited 198, 154, 140, 116, 107, 62, and 12 participants, respectively, totaling 789 PwD. Of the included, 44% (*n* = 347) had facial dystonia/dyskinesia (blepharospasm [BPS] and HFS), 43% (*n* = 339) had cervical dystonia (CD), 5.8% (*n* = 46) had oromandibular dystonia (OMD), 5.4% (*n* = 43) had SegD, and 1.5% (*n* = 12) had task‐specific dystonia (TSD). The mean age was 57 (± 14.8; range 18–90), and 66.8% of the patients were female. They had a mean of 7.9 years (± 4.8) of education, with the highest in TSD. PwD with BPS/HFS were significantly less educated compared to other types of dystonia (one‐way ANOVA *F*(2780) = 40.94; *p* < 0.001). Almost half of our patients were housewives (*n* = 379; 48%), and 28% (*n* = 220) were employed. The remaining were retired (18.8%; *n* = 148), unemployed (3%; *n* = 24), and students (1.5%; *n* = 12). Around 70% were in the very low or low‐income group (₺0–10,000 [Turkish Liras], monthly), and only 6.8% reported a monthly household income higher than ₺20,000. Demographic information for each diagnostic category is given in Table 1.

**TABLE 1:** Demographics, challenges, and burden during the diagnostic stage presented for each diagnostic category.

	BPS/HFS (*n* = 347)	CD (*n* = 339)	OMD (*n* = 46)	SegD (*n* = 43)	TSD (*n* = 12)
Demographic and disease‐related information					
Age, years, mean (SD)	62.3 (12.4)	54.5 (13.9)	55.4 (14.6)	37.9 (15.2)	48.5 (19.3)
Female sex, % (*n*)	66.6 (231)	71.4 (242)	73.9 (34)	39.5 (17)	16.7 (2)
Education, years, mean (SD)	6.7 (4.8)	8.8 (4.7)	8.4 (4.5)	9.0 (4.1)	11.1 (4.3)
Most frequent occupation, %	Hw (54.8)	Hw (44.0)	Hw (52.2)	Hw (32.6)	Empl (75.0)
Most frequent household income, %	Low (39.2)	Low (39.2)	Low (41.3)	Low (39.5)	Middle (25.0)
Disease duration, yrs, median (range)	8 (0.5–33)	7 (1–40)	4.5 (1–24)	14 (1–48)	5 (0.5–20)
Symptom duration, yrs, median (range)	10 (1–35)	10 (1–47)	7 (1–24)	18 (2–48)	7.5 (1–21)
Challenges until diagnosis					
How many visits, median (IQR)	2 (2)	2 (2)	3 (2.25)	2 (3)	2 (1.75)
How many specialties, median (IQR)	1 (1)	1 (1)	2 (1)	1 (1)	1 (1)
Delay in diagnosis, yrs, median (range)	0.75 (2)	1 (4)	1 (3)	1 (3)	0.75 (2.75)
Initial incorrect diagnosis, % (*n*)	10.4 (36)	22.1 (75)	21.7 (10)	2.3 (1)	16.7 (2)
The most frequent incorrect diagnosis	Dry eye	Functional	Functional	Spasticity	CTS
Travelled to another city for diagnosis, % (*n*)	25.1 (87)	33.6 (114)	43.5 (20)	37.2 (16)	41.7 (5)
Applied to private clinic for diagnosis, % (*n*)	34.6 (120)	49 (166)	50 (23)	46.5 (20)	50 (6)
Burden until diagnosis					
Burdensome, % (*n*)	54.2 (188)	65.8 (223)	80.4 (37)	76.7 (33)	58.3 (7)
Partially burdensome, % (*n*)	29.7 (103)	20.1 (68)	10.9 (5)	16.3 (7)	33.3 (4)
Not burdensome, % (*n*)	15.3 (53)	13.3 (45)	8.7 (4)	7.0 (3)	8.3 (1)

Abbreviations: BPS/HFS, blepharospasm/hemifacial spasm; CD, cervical dystonia; CTS, carpal tunnel syndrome; Empl, employed; Hw, housewife; IQR, interquartile range; OMD, oromandibular dystonia; SD, standard deviation; SegD, segmental dystonia; TSD, task‐specific dystonia; yrs, years.

### 3.1 Diagnostic Burden

On average, PwD reported a disease duration of 7 years, with a median of around 1 year delay in diagnosis for each dystonia type. Around 15% of all PwD reported that they initially received incorrect diagnoses, the most frequent of which were functional/psychogenic (28.2%), cervical problems (disc herniation, cervical kyphosis, etc.; 17.7%), and dry eye (12.1%). Initial incorrect diagnosis was reported mostly in PwD with CD (22.1%) and OMD (21.7%) with the least in SegD (2.3%; χ^2^; *p* < 0.0001). Additionally, 75.2% of the PwD reported receiving a diagnosis after visiting no more than two different specialties and required a median of two visits.

Of all PwD, 30.7% (*n* = 242) reported that they traveled to another city for diagnostic purposes. We performed a logistic regression to identify factors associated with the need to travel to another city, which showed a good fit and correctly classified 70% of the cases (Nagelkerke *R* ^2^ = 7.2%; χ^2^(18) = 40.197; *p* = 0.002). The model showed that older PwD were slightly less likely to travel to another city (OR = 0.98; 95% CI: 0.97–0.99; *p* = 0.008). Furthermore, being male (OR = 2.01; 95% CI: 1.27–3.35; *p* = 0.003), symptom duration (OR = 1.03; 95% CI: 1.00–1.05; *p* = 0.028), and having an initial misdiagnosis (OR = 1.60; 95% CI: 1.03–2.47; *p* = 0.035) were associated with traveling to another city. Also, compared to BPS/HFS, PwD with OMD were twice as likely to travel for diagnostic purposes (OR = 2.16; 95% CI: 1.11–4.20; *p* = 0.023). PwD with a higher income were also more likely to travel (OR = 2.03; 95% CI: 1.03–4.00; *p* = 0.041). Education, occupation, and delay in diagnosis were not significantly associated (Table S1). In addition, 42.6% (*n* = 336) of PwD reported an application to a private clinic to receive a diagnosis. The regression model for the prediction of application to a private clinic explained 12.1% of the variance in group membership and also showed an improvement with covariates (χ^2^(18) = 72.487; *p* < 0.001). PwD with an initially incorrect diagnosis were 3.8 times more likely to apply to a private center compared to those with a correct diagnosis (OR = 3.78; 95% CI: 2.42–5.90; *p* < 0.001). Also, an increase in education was slightly related to private clinic applications (OR = 1.05; 95% CI: 1.01–1.10; *p* = 0.021). Other factors were not significant predictors of private center application (Tables 1 and S2). Moreover, 78.3% of PwD stated that they were adequately informed about their condition following the diagnosis.

The period between the onset of the symptoms and diagnosis was reported as burdensome by 62% of the patients. To identify the factors associated with the diagnostic burden that PwD felt, an ordinal logistic regression using a generalized linear model (GLM) was constructed. The full model was significantly improved in fitness over the null model (χ^2^(18) = 134.644; *p* < 0.0001). The GLM model revealed that each year's decrease in age and education increased the odds of feeling burden by 2% (95% CI: 0.96–0.99; *p* < 0.001) and 5% (95% CI: 0.91–1.0; *p* < 0.038), respectively. Delay in diagnosis was also associated with burden (OR = 1.13; 95% CI: 1.05–1.21; *p* = 0.001). In addition, PwD with misdiagnosis and who applied to a private center were 2.1 (95% CI: 1.22–3.64; *p* = 0.007) and 2.3 (95% CI: 1.67–3.33; *p* < 0.001) times more likely to experience diagnosis‐related burden, respectively, compared to ones who were correctly diagnosed or applied to government‐funded healthcare facilities. Other included factors were not significant predictors of diagnostic burden (Table S3). The vast majority of the PwD were diagnosed by a neurologist (93.5%), and most PwD reported that they were adequately informed about their condition by their physician. Details of the results are given in Table 1 and Supporting Information.

### 3.2 Treatment Burden

Table 2 summarizes the burden of the PwD at the stage of BoNT treatment. Unlike the diagnostic stage, challenges in treatment access and regular follow‐ups did not differ significantly between the types of dystonia; therefore, the data are presented as a whole.

**TABLE 2:** Treatment burden in patients receiving BoNT.

Were are you adequately informed by your doctor regarding the BoNT treatment? % (*n*)	
Yes	80.6 (636)
Partially	10.5 (83)
No	4.8 (38)
Not remember/no comment	3.9 (31)
How difficult is it for you to find an appointment for BoNT treatment? % (*n*)	
Not difficult	68.7 (542)
Sometimes difficult	16.6 (131)
Difficult	12.4 (98)
Very difficult	2.2 (17)
Where do you come for the BoNT treatment? % (*n*)	
City center	57.8 (456)
City districts/villages	16.6 (131)
Another city	25.6 (202)
How much do you spend to receive BoNT treatment? median (range)	
City districts	₺200 (20–1200)
Another city	₺1000 (40–15000)
What kind of organization do you need to come to BoNT treatment? % (*n*)	
Not organization required	42.6 (336)
Arrange an accompanying person	25.9 (204)
Arrange travel	22.4 (117)
Take a leave from school/job	17.7 (140)
Find an accommodation	5.3 (42)
Arrange a babysitter	4.1 (32)
Arrange a caregiver for pets	1.8 (14)
Other	3.3 (26)
Can you go to your appointments as advised? % (*n*)	
Yes	85.3 (673)
No	14.6 (115)
Reason for not going % (*n*)	
Cannot find an appointment	6.3 (50)
Because it is in another city—cannot afford	1.4 (11)
Because it is in another city—cannot find the time	1.6 (13)
BoNT does not work	0.3 (2)
Cannot find an accompanying person	0.6 (5)
Other	5.7 (45)
Private hospital/clinic for the BoNT treatment, % (*n*)	24.5 (193)
Satisfied with the BoNT treatment? % (*n*)	
Very satisfied	49.8% (393)
Satisfied	44.1% (348)
Somewhat satisfied	4.9% (39)
Not satisfied	0.9% (7)
Alternative medicine, % (*n*)	11 (87)
Cupping	53.5 (46)
Acupuncture	31.4 (27)
Leech therapy	23.3 (20)
Ozone therapy	9.3 (8)
Phytotherapy	5.8 (5)
Other	19.8 (17)
Benefit from alternative medicine, % (*n*)	
No benefit	73.3 (55)
Partial benefit	21.3 (16)
Much improved	5.3 (4)

Abbreviation: BoNT, botulinum neurotoxin.

**FIGURE 1:**
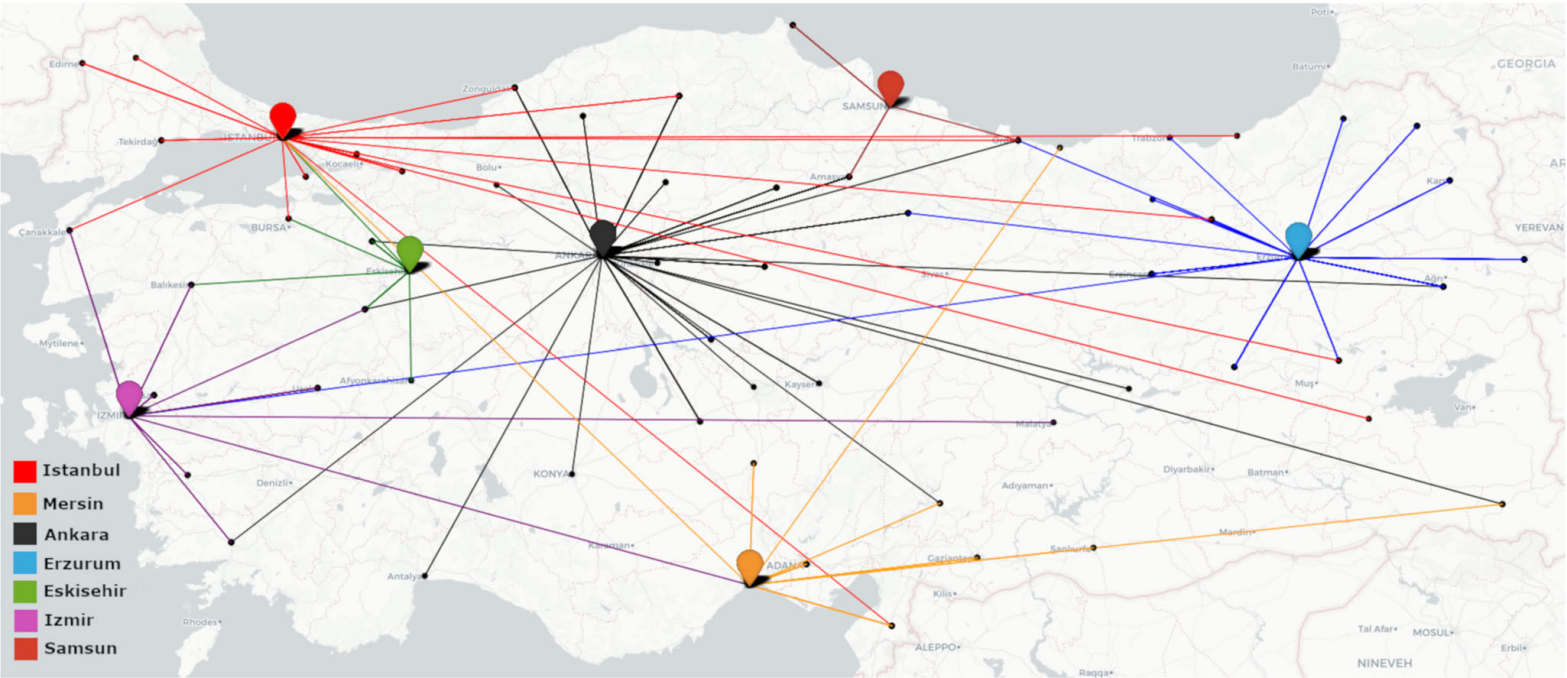
Map of Turkey showing where the participating movement disorder units accept patients from.

Participating PwD reported that they were adequately informed about the effects and side effects of the BoNT treatment before the injection. They received their injections usually within 1 month following diagnosis. Most PwD received injections every 3 months. Of all PwD, 69% reported that they easily found an appointment for the BoNT treatment. The data also showed that the participating (seven) movement disorder centers received PwD from 58 cities across Turkey, which makes 25.6% of the treated PwD (Figure 1). Because of the absence or limited availability of BoNT/dystonia clinics in their hometown, these PwD traveled every 3 months a median of 216 km (38–1643) and spent a median of ₺1000, which is equal to 1/5 of the monthly income of 32.7% and 1/10 of 40.6% of PwD.

A quarter of the PwD reported applying to a private clinic and paying to receive BoNT treatment (Table 2). The logistic regression model to determine the factors related to this was successful with included predictors (Nagelkerke *R* ^2^ = 8.6%; χ^2^(14) = 45.005; *p* < 0.001). The model showed that compared to BPS/HFS, patients with CD were 1.65 times more likely to report having an injection in a private clinic (95% CI: 1.13–2.342; *p* = 0.010). Moreover, compared to the lowest income group, each level of income had a significant likelihood of private clinic application, tripling in the highest income group (OR = 3.16; 95% CI: 1.51–6.63; *p* = 0.002). Additionally, PwD who were not satisfied with the BoNT treatment were 2.32 times more likely to have applied to a private clinic (95% CI: 1.22–4.44; *p* = 0.011). Finally, compared to PwD living in city centers, those coming from another city also reported a higher likelihood of application to private healthcare centers for BoNT injections (OR = 1.75; 95% CI: 1.18–2.60; *p* = 0.005). Details of the regression statistics are given in the Table S4.

In the survey, PwD were also asked what kind of organization they needed to be able to receive the treatment. While 42.6% stated no organization was required, 22.4% and 25.9% reported that they need to arrange travel and an accompanying family member, respectively. Additionally, 17.7% needed an official leave from work/school. Despite these difficulties, 85.3% were able to come to their appointments as advised, and 94.3% thought that their physician spent enough time discussing the treatment in controls.

**FIGURE 2:**
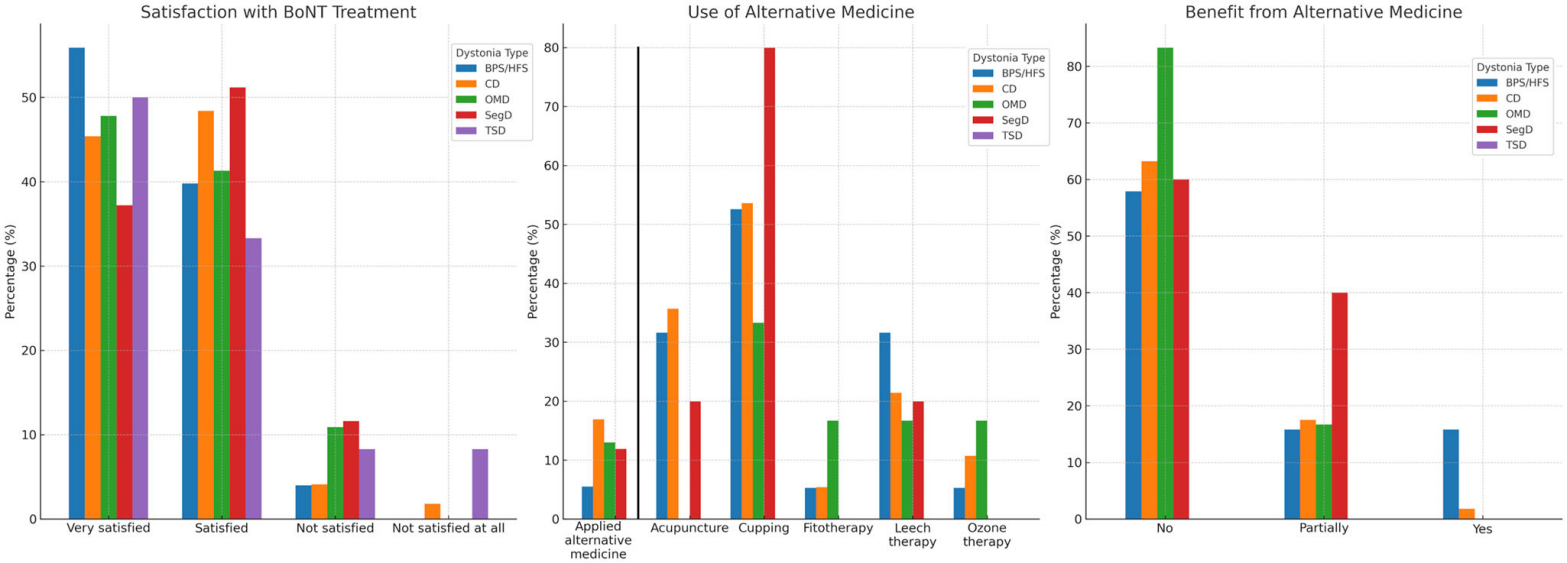
Satisfaction from the BoNT and using alternative medicine in types of dystonia (the percentage values given for alternative treatment options represent the percentages among those who used alternative treatments).

In the study, more than 90% reported that they were very satisfied or satisfied with the effect of the BoNT treatment. However, significant differences were found in satisfaction levels between the types of dystonia in the logistic regression model with the level of satisfaction (dichotomized as satisfied vs. unsatisfied) as the dependent variable and age, sex, education, type of dystonia, having BoNT injections in a private center or not, history of alternative medicine, and the frequency of injections. The predictors significantly improved the regression model (Nagelkerke *R* ^2^ = 7.7%; χ^2^(10) = 21.368; *p* = 0.019). The model showed that the satisfaction from the BoNT treatment was significantly affected by the type of dystonia and (as mentioned above) history of having BoNT injections in a private clinic. Compared to BPS/HFS, PwD with OMD were 3.34 (95% CI: 1.10–10.3; *p* = 0.033) times less likely to be satisfied with BoNT treatments. Also, the odds of being unsatisfied from the BoNT treatment were around four times (OR = 3.92; 95% CI: 1.11–13.7; *p* = 0.033) and nine times (OR = 8.96; 95% CI: 1.54–52.0; *p* = 0.015) increased for SegD and TSD, respectively. The level of satisfaction was not significantly different in CD, compared to BPS/HFS (OR = 1.45; 95% CI: 0.67–3.15; *p* = 0.340). Apart from that, PwD with a history of application to a private clinic for BoNT injections were twice as likely to be unsatisfied with their treatment (OR = 2.26; 95% CI: 1.18–4.32; *p* = 0.013). Other factors were not significant predictors (Figure 2 and Table S5).

Our survey also included questions on alternative medicine. In total, 11% (*n* = 87) of the participating PwD reported that they received alternative approaches. The most frequent methods used were cupping and acupuncture with a median of two sessions (1–104). These methods were significantly more frequently received by patients with CD (16.9%), SegD (11.9%), and OMD (13%), with the least in facial dystonia (BPS/HFS, 5.5%) and TSD (0%; chi‐square, *p* < 0.001). The majority of the patients reported no benefit from these approaches (Tables 2 and S6; Figure 2).

## 4 Discussion

In this study, we attempted to explore the challenges of PwD during the stages of diagnosis and BoNT treatment. Our results showed significant burdens in both stages, which were not documented previously in this population.

Overall, PwD reported a diverse range of age of onset which was in line with the reported literature (O'Riordan et al. 2004; Defazio et al. 2007). The majority of PwD with BPS/HFS, CD, and OMD were female, whereas SegD and TSD were predominantly male. On average, PwD had low education level (around 8 years) and were most frequently housewives in contrast to Western societies (C. Comella et al. 2021; Defazio et al. 2017). They also reported a low household income except for TSD, who had a higher education and income and were more educated.

### 4.1 Burden in the Diagnostic Stage

Our survey on the difficulties of the Turkish PwD until reaching diagnosis showed challenges in several steps. First, in each type of dystonia, diagnosis was delayed approximately 1 year. Within this period, they reported a median of two doctor visits (three for OMD) of one specialty (two for OMD), which was neurology. While a 1‐year delay may seem reasonable and is less than in Western data (Smit et al. 2021; C. Comella et al. 2021; Defazio et al. 2017), it also reflects the current absence of a healthcare referral chain in Turkey, meaning that PwD could book an appointment with a neurologist without the requirement of a referral, which prevented delays in specialist care. Likewise, the reported 15% misdiagnosis rate (with neurologists) is much less than previous reports with family doctors reaching 50% (Benson et al. 2022). This accessibility, however, comes at a price as the increased patient load on specialists may lead to less detailed examinations, evidenced by a 15% misdiagnosis rate, which is high for neurologists, and a high rate of reports of travel to another city and application to private clinics (Table 1). The most frequent incorrect diagnosis was “dry eye” in PwD with facial dystonia, “functional/psychogenic” in CD and OMD, and “spasticity” in SegD. No inaccurate diagnosis was noted for TSD, possibly because the phenomenology of TSD is unique and is unlike any other condition.

The results showed that one in four patients with BPS/HFS traveled to another city to receive a diagnosis for their symptoms. This rate was even higher for CD and SegD and were highest in PwD having OMD or TSD. Moreover, in all dystonia types, one in two or three PwD had to consult private clinics and pay to get a diagnosis. Again, an initially incorrect diagnosis was the most successful predictor for private clinic application with odds of 3.8. The outcomes of these regression models indicate that mobilization to another city and/or application to private clinics constitute a considerable part of the diagnostic journey of Turkish PwD, especially for the ones with an initial incorrect diagnosis. Interestingly, monthly income was not a significant predictor for travel to another city or private clinic applications. This indicates that PwD might have been compelled to travel or spend money to reach an accurate diagnosis regardless of their income. Around three out of four PwD stated they were adequately informed following their diagnosis. This rate is much higher than the published patient map in Europe and the UK (Benson et al. 2022) but should be interpreted with caution considering the potential overreporting due to the face‐to‐face conduction of the survey.

Overall, 50%–80% of the PwD reported that the diagnostic period was burdensome, which was highest in PwD with OMD. Also, the ones with a delay in diagnosis and younger or less educated PwD reported more hardships during the diagnostic process. Among the significant contributors to the diagnostic burden of PwD, private clinic applications had the biggest share, doubling the burden. This is plausible given the low household income in the majority of PwD. Also, as expected, the initial incorrect diagnosis was associated with an increased diagnostic burden. These results underscore the necessity of organizing educational activities for local neurologists, which could prevent incorrect diagnoses, reduce expenses for travel and private clinic applications of PwD and, thus, lessen the burden until diagnosis (Centen et al. 2023).

### 4.2 Burden in the BoNT Treatment Stage

The data showed that most PwD could receive their first injections immediately after diagnosis, in parallel with other countries (C. Comella et al. 2021). In addition, around two‐thirds of all PwD reported that finding an appointment for BoNT injections was not difficult. Having said that, arriving at an appointment seems to be accompanied by hardships for some PwD, who had to travel to reach the treatment. One in four PwD of the participating tertiary centers came from another city (Figure 1). Similar to the diagnostic stage, these patients had to travel to access BoNT every 3–4 months and spend up to one‐third of their monthly household income on each visit. This cost is by no means reasonable and adds to the challenges of the PwD. Furthermore, additional arrangements such as booking the best timing for travel, arranging an accompanying person, taking a leave from work, finding accommodation, etc. were part of the treatment process, further complicating the access to treatment (Table 2). Most PwD stated that they could go to their appointments as advised. However, the data show that this was achieved with much effort for some PwD.

Most patients receive treatment at the participating centers, but a quarter of PwD reported paying for their BoNT treatment at a private clinic at least once. The data revealed that (compared to BPS/HFS) PwD with CD, PwD who were unsatisfied with their BoNT treatment, those with a higher income, and those coming from another city were more likely to report private clinic applications. Still, around 95% of all PwD reported satisfaction with their BoNT injections, similar to previous reports (Sethi et al. 2012; Colosimo, Charles et al. 2019; Trosch et al. 2020). Nonetheless, satisfaction from the BoNT treatment differed within the types of dystonia. While no significant difference was found between BPS/HFS and CD, PwD with OMD were three times, PwD with SegD were four times, and PwD with TSD were nine times more likely to be unsatisfied with their treatment, compared to BPS/HFS (Table S5). This is also in line with the literature (C. L. Comella 2018; Marciniec et al. 2020; Dadgardoust et al. 2019; Zakin and Simpson 2021), reflecting the contrast within the types of dystonia in terms of treatment.

This study also investigated the practices of PwD regarding complementary and alternative treatment. Around 11% of the PwD confirmed trying an alternative treatment approach in the name of traditional or holistic medicine, which was much less than the reported rates in the German population (Junker et al. 2004; Fleming et al. 2012; Viehmann et al. 2014). The most frequently applied treatment was cupping and acupuncture followed by leech therapy. Consistent with the responses for satisfaction from the BoNT treatment, BPS/HFS was the group that reported an alternative treatment the least with 5.5%. Independent of the type of dystonia, the majority of the PwD reported no or less benefit from these treatment modalities similar to the previous reports (Junker et al. 2004; Viehmann et al. 2014; Figure 2 and Table S6). With regard to the effect of alternative approaches, while some small scale or case studies reported favorable outcomes (Bega et al. 2018; Bao et al. 2019; Horibe et al. 2024), the existing literature is mostly comprised of low quality studies hindering to draw definite conclusions (Gong et al. 2022).

Limitations of our study have to be mentioned. First and most importantly, the data were collected from PwD who were regularly treated in our clinics. The opinions of PwD who had not been diagnosed or treated or the ones who could not find an appointment or were unable to come were out of access, indicating a sampling bias that is intrinsic to all survey studies. Moreover, a response bias was also possible, especially in questions about whether the patients were adequately informed or whether they were satisfied with the BoNT treatment. PwD might have felt compelled to a more positive answer in the face‐to‐face survey, compared to surveys that were mailed (Benson et al. 2022). In that context, reports on the alternative treatment might have been downplayed by PwD with thoughts of inappropriateness in the presence of healthcare personnel. A recall bias was also unavoidable regarding the questions related to the diagnostic process. Also, access to other modalities including physical or occupational therapies, psychological support, or deep brain stimulation was excluded from the survey and should be evaluated with a wider population including PwD with generalized dystonia (Benson et al. 2022). Furthermore, data on some types of dystonia, especially the TSD, should be interpreted with caution considering the smaller sample size. Apart from that, the large representative sample size, collecting data of PwD from different regions, the application of the survey in a standardized setting face‐to‐face by medical personnel, and the detailed questioning of both diagnostic and treatment processes are the clear strengths of the current study. To our knowledge, this is the largest study investigating patient burden with data obtained directly from a firsthand patient perspective in a non‐Western population.

**FIGURE 3:**
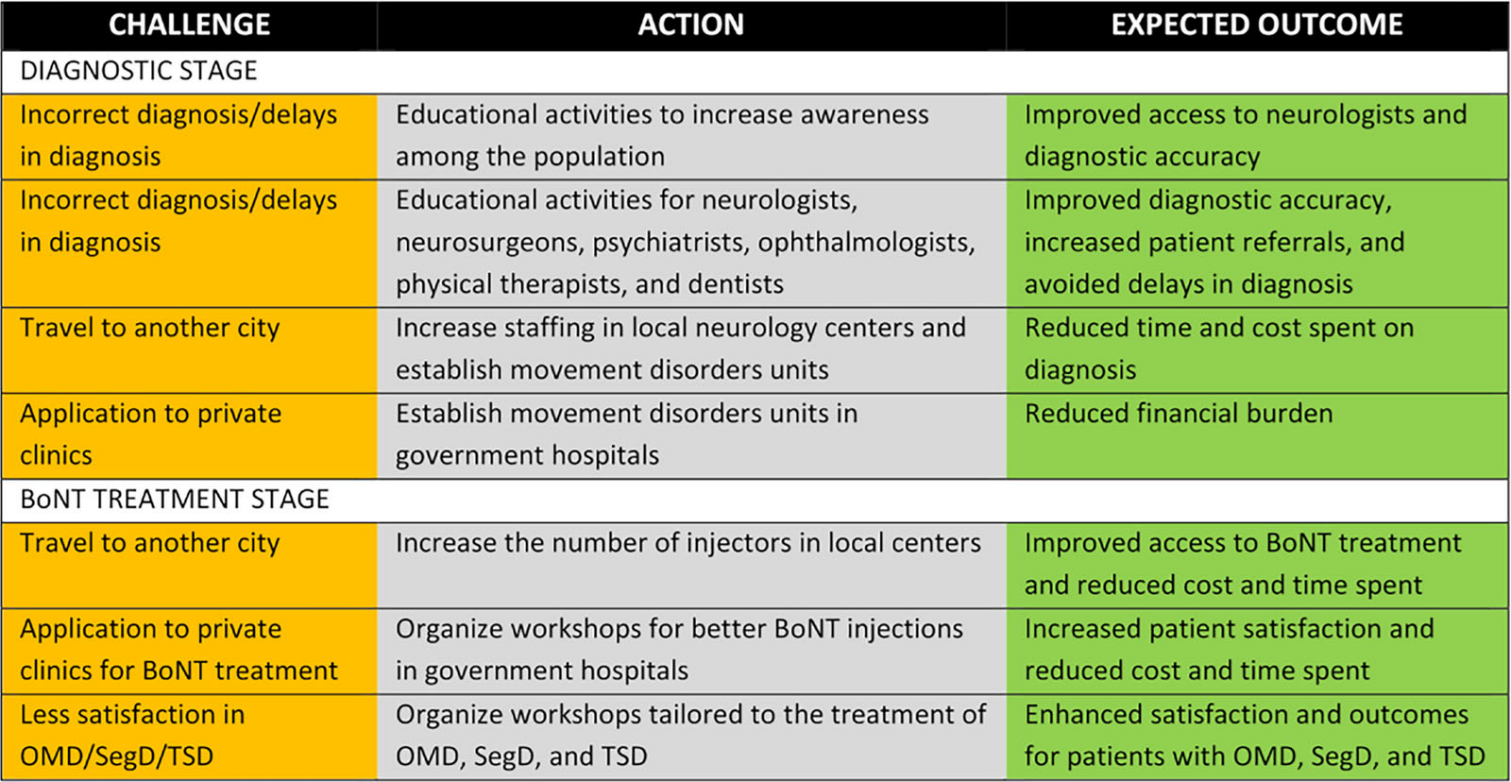
Challenges, actions, and expected outcomes in diagnostic and BoNT treatment stages of PwD.

In conclusion, we believe our findings highlight the unmet needs of PwD in terms of diagnosis and BoNT treatment (Figure 3). The majority of Turkish PwD reported that the diagnostic stage was burdensome and was complicated by incorrect diagnoses and the need to travel to another city or apply to private clinics for diagnosis. At the treatment stage, many PwD had to travel to another city to receive BoNT injections regularly, which cost them considerable time and a share of their monthly income. Additionally, PwD with OMD, SegD, and TSD were not as satisfied as facial dystonia or CD. Based on this data an action plan to relieve these challenges has been drawn (Figure 2). While these results were obtained from Turkish PwD, they may also mirror challenges in other countries with similar cultural or economic state, where no data are available. Overall, the findings reveal the gaps in healthcare in PwD and point to a necessity for resource utilization policies and educational activities, which could facilitate correct diagnoses and better treatment of individuals with dystonia.

## Author Contributions

**Rezzak Yilmaz**: conceptualization, investigation, writing–original draft, formal analysis, project administration, methodology. **Nevra Öksüz**: investigation, writing–review and editing, formal analysis. **Mustafa Ceylan**: investigation, writing–review and editing, conceptualization. **Bedia Samanci**: conceptualization, investigation, writing–review and editing. **Ahmet Acarer**: conceptualization, investigation, writing–review and editing. Nazlı Durmaz Çelik: conceptualization, investigation, writing–review and editing. **Hacer Erdem**: conceptualization, investigation, writing–review and editing. **Serhat Özkan**: conceptualization, investigation, writing–review and editing, supervision. **Haşmet Hanağası**: conceptualization, investigation, writing–review and editing, supervision. **Okan Dogu**: conceptualization, investigation, writing–review and editing, supervision. **M. Cenk Akbostancı**: conceptualization, investigation, writing–review and editing, supervision, resources, methodology.

### Peer Review

The peer review history for this article is available at https://publons.com/publon/10.1002/brb3.70325

## Supporting information



Supporting Information

## Data Availability

The data that support the findings of this study are available on request from the corresponding author. The data are not publicly available due to privacy or ethical restrictions.
