# Supplementary material for: Patient Burden in Dystonia Diagnosis and Botulinum Toxin Treatment: A Nationwide Survey in Turkey
Source: Brain Behav. 2025 Feb 28;15(3):e70325. doi: 10.1002/brb3.70325 (PMC11870789; doi:10.1002/brb3.70325)
Supplement: Supplementary file 1 — Supporting Information [file BRB3-15-e70325-s001.docx]

Supplementary file for:

Patient Burden in Dystonia Diagnosis and Botulinum Toxin Treatment:

A Nationwide Survey in Turkey

Rezzak Yilmaz^1,2^, Nevra Öksüz^3^, Mustafa Ceylan^4^, Bedia Samanci^5^, Ahmet Acarer^6^, F. Nazlı Çelik^7^, Hacer Erdem^8^, Serhat Özkan^7^, Haşmet Hanağası^5^, Okan Dogu^3^, M. Cenk Akbostancı^1,2^

1. Ankara University School of Medicine Department of Neurology
2. Ankara University, Brain Research Center
3. Mersin University School of Medicine Department of Neurology
4. Atatürk University School of Medicine Department of Neurology
5. Istanbul University School of Medicine Department of Neurology
6. Ege University School of Medicine Department of Neurology
7. Osmangazi University School of Medicine Department of Neurology
8. Ondokuz Mayıs University School of Medicine Department of Neurology

**Supplementary file**

**Methods,**

**Survey:**

Patient no:

Gender:

Age:

City of residence:

Years of education completed:

Occupation:

Total household income:

- ₺0 – 5,000
- ₺6 – 10,000
- ₺11 – 15,000
- ₺16 – 20,000
- ₺20,000 and above

Primary type of dystonia:

- Blepharospasm / Hemifacial spasm
- Cervical dystonia
- Oromandibular dystonia
- Segmental dystonia
- Task-specific dystonia

BURDEN AT DIAGNOSIS

1. How many years ago did your first symptoms begin?
2. How many years ago were you diagnosed?
3. Before your diagnosis, how many times and to how many different doctors did you go for these symptoms? What were their specialties? (neurology, orthopedics, dentist, physical therapy, etc.) (Please also indicate multiple visits to the same doctor).
4. Did you change cities for diagnosis?
   1. Yes
   2. No
5. Did you receive any other diagnoses different from your current diagnosis?
   1. Yes, what were they? (...)
   2. No
6. What tests were conducted on you until your current diagnosis was made?
7. Did you receive any treatment for a different diagnosis?
   1. Yes, what were they? (...)
   2. No
8. Did you visit a private clinic or hospital for diagnosis?
   1. Yes
   2. No
9. Was the period from the onset of symptoms to diagnosis difficult and challenging?
   1. Yes
   2. Partly
   3. No
10. Which specialty did the diagnosing doctor belong to?
11. Do you think the doctor who diagnosed you spent enough time explaining your disease?
    1. Yes
    2. Partly
    3. No

BURDEN AT BONT TREATMENT

1. Do you think your doctor spent enough time explaining the BoNT treatment when you first started?
   1. Yes
   2. Partly
   3. No
2. How often do you go for check-ups? (average number in months)
3. How difficult is it to find an appointment for BoNT treatment?
   1. I can easily find it
   2. Sometimes easy, sometimes difficult
   3. I have difficulties
   4. I can hardly find it
4. Where do you come from for botox treatment?
   1. City center
   2. Districts or villages of the city
   3. Outside the city
5. Which district or city? (This question will be asked to everyone except those coming from within the city)
6. What transportation do you use to come for BoNT treatment? (This question will be asked to everyone except those coming from within the city)
   1. Intercity bus/district minibus
   2. By plane
   3. By my private vehicle
   4. By taxi
   5. By train
7. How much does it cost you on average to travel to here to receive BoNT treatment? (Include round-trip bus/plane fare, hotel costs if staying, babysitter fees, etc.) (This question will be asked to everyone except those coming from within the city)
8. What do you need to organize for botox treatment?
   1. No organization needed
   2. I need to arrange transportation because I live in another city
   3. I need to arrange accommodation because I live in another city
   4. I need to take leave from work/school
   5. I need to arrange a babysitter for my child
   6. I need to arrange suitable conditions for my pet
   7. Other (…)
9. Can you always attend the treatment as recommended?
   1. Yes
   2. No
10. If not, why can’t you attend?
    1. I have difficulties having an appointment
    2. Financial difficulties due to being in a different city
    3. Difficulties allocating time due to being in a different city
    4. I do not want to go because I think the treatment is ineffective
    5. Other (…)
11. Do you think your doctor spends enough time with you during check-ups?
    1. Yes
    2. Partly
    3. No
12. Did you ever visit a private clinic or hospital for BoNT treatment?
    1. Yes
    2. No
13. Are you satisfied with the BoNT treatment?
    1. Very satisfied
    2. Satisfied
    3. Not very satisfied
    4. Not satisfied at all
14. Did you ever try alternative medicine for your complaint? (Cupping, leech therapy, talismans, acupuncture, ozone therapy, etc.)
    1. Yes, what and how many times (...)
    2. No
15. If you tried, did you find it beneficial?
    1. Yes
    2. Partly
    3. No
16. Looking back, what would you like to be different about your diagnosis/treatment process? What would have helped you?

**Results**

Supp-table-1: Parameter estimates for the logistic regression analysis to detect factors associated with traveling to another city for diagnosis.

|  | **B** | **S.E.** | **Wald** | **df** | **Sig.** | **Exp(B)** | **95% CI** | |
| --- | --- | --- | --- | --- | --- | --- | --- | --- |
| Age | -.020 | .007 | 6.997 | 1 | **.008** | .981 | .966 | .995 |
| Male sex | .726 | .247 | 8.642 | 1 | **.003** | 2.067 | 1.274 | 3.354 |
| Education (years) | -.020 | .023 | .697 | 1 | .404 | .981 | .937 | 1.027 |
| Occupation (housewife) |  |  | 3.787 | 4 | .436 |  |  |  |
| Occupation (employed) | -.535 | .288 | 3.463 | 1 | .063 | .586 | .333 | 1.029 |
| Occupation (retired) | -.393 | .312 | 1.585 | 1 | .208 | .675 | .366 | 1.244 |
| Occupation (unemployed) | -.437 | .537 | .662 | 1 | .416 | .646 | .225 | 1.852 |
| Occupation (student) | -.065 | .667 | .010 | 1 | .922 | .937 | .253 | 3.466 |
| Income 0-5000₺ |  |  | 4.222 | 4 | .377 |  |  |  |
| Income 6-10000₺ | .169 | .203 | .691 | 1 | .406 | 1.184 | .795 | 1.762 |
| Income 11-15000₺ | .173 | .271 | .406 | 1 | .524 | 1.189 | .699 | 2.023 |
| Income 16-20000₺ | .706 | .346 | 4.173 | 1 | .**041** | 2.026 | 1.029 | 3.990 |
| Income >20000₺ | .307 | .381 | .649 | 1 | .421 | 1.360 | .644 | 2.871 |
| Symptom duration | .026 | .012 | 4.836 | 1 | .028 | 1.026 | 1.003 | 1.050 |
| Delay in diagnosis | .005 | .021 | .054 | 1 | .816 | 1.005 | .964 | 1.047 |
| Incorrect diagnosis | .468 | .222 | 4.448 | 1 | **.035** | 1.597 | 1.034 | 2.469 |
| Type of dystonia (BPS/HFS) |  |  | 7.132 | 4 | .129 |  |  |  |
| Type of dystonia (CD) | .302 | .186 | 2.621 | 1 | .105 | 1.352 | .938 | 1.948 |
| Type of dystonia (OMD) | .771 | .339 | 5.168 | 1 | **.023** | 2.161 | 1.112 | 4.199 |
| Type of dystonia (SegD) | -.116 | .412 | .080 | 1 | .778 | .890 | .397 | 1.998 |
| Type of dystonia (TSD) | .401 | .636 | .397 | 1 | .529 | 1.493 | .429 | 5.191 |
| Constant | -.322 | .527 | .373 | 1 | .542 | .725 |  |  |
| Dependent Variable: Travel to another city for diagnosis. Predicted probability is for “Travel to another city= yes”. | | | | | | | | |

Supp-table-2: Parameter estimates for the logistic regression analysis to detect factors associated with application of private clinics for diagnosis.

|  | **B** | **S.E.** | **Wald** | **df** | **Sig.** | **Exp(B)** | **95% CI** | |
| --- | --- | --- | --- | --- | --- | --- | --- | --- |
| Age | -.004 | .007 | .360 | 1 | .548 | .996 | .982 | 1.010 |
| Male sex | .102 | .227 | .201 | 1 | .654 | 1.107 | .710 | 1.726 |
| Education (years) | .051 | .022 | 5.312 | 1 | **.021** | 1.052 | 1.008 | 1.099 |
| Occupation (housewife) |  |  | .655 | 4 | .957 |  |  |  |
| Occupation (employed) | -.128 | .268 | .229 | 1 | .632 | .880 | .520 | 1.487 |
| Occupation (retired) | -.168 | .289 | .339 | 1 | .560 | .845 | .480 | 1.489 |
| Occupation (unemployed) | -.275 | .531 | .268 | 1 | .605 | .760 | .268 | 2.151 |
| Occupation (student) | .134 | .677 | .039 | 1 | .843 | 1.144 | .303 | 4.313 |
| Income 0-5000₺ |  |  | 1.651 | 4 | .800 |  |  |  |
| Income 6-10000₺ | .153 | .193 | .631 | 1 | .427 | 1.166 | .798 | 1.702 |
| Income 11-15000₺ | .282 | .256 | 1.219 | 1 | .270 | 1.326 | .804 | 2.188 |
| Income 16-20000₺ | .321 | .337 | .904 | 1 | .342 | 1.378 | .712 | 2.669 |
| Income >20000₺ | .279 | .363 | .589 | 1 | .443 | 1.321 | .649 | 2.691 |
| Symptom duration | .006 | .011 | .275 | 1 | .600 | 1.006 | .984 | 1.029 |
| Delay in diagnosis | -.005 | .022 | .057 | 1 | .811 | .995 | .954 | 1.038 |
| Incorrect diagnosis | 1.330 | .227 | 34.254 | 1 | **.000** | 3.779 | 2.421 | 5.899 |
| Type of dystonia (BPS/HFS) |  |  | 3.789 | 4 | .435 |  |  |  |
| Type of dystonia (CD) | .317 | .175 | 3.294 | 1 | .070 | 1.373 | .975 | 1.934 |
| Type of dystonia (OMD) | .380 | .338 | 1.262 | 1 | .261 | 1.462 | .754 | 2.836 |
| Type of dystonia (SegD) | .333 | .389 | .729 | 1 | .393 | 1.394 | .650 | 2.991 |
| Type of dystonia (TSD) | .198 | .633 | .098 | 1 | .754 | 1.219 | .353 | 4.213 |
| Constant | -1.034 | .504 | 4.212 | 1 | .040 | .356 |  |  |
| Dependent Variable: Private clinic application for diagnosis. Predicted probability is for “Private clinic application= yes”. | | | | | | | | |

Supp-table-3: Parameter estimates for the general linear model ordinal regression analysis to detect factors associated with diagnostic burden.

| **Parameter** | | **B** | **Std. Error** | **95% Wald CI** | | **Hypothesis Test** | | | **Exp(B)** | **95% Wald CI for Exp(B)** | |
| --- | --- | --- | --- | --- | --- | --- | --- | --- | --- | --- | --- |
|  |  |  |  | **Lower** | **Upper** | **Wald Chi-sq** | **df** | **Sig.** |  | **Lower** | **Upper** |
| Threshold | [Diagnostic burden=no] | -2.372 | 1.1285 | -4.584 | -0.160 | 4.417 | 1 | 0.036 | 0.093 | 0.010 | 0.852 |
|  | [Diagnostic burden =mild] | -0.873 | 1.1247 | -3.077 | 1.332 | 0.602 | 1 | 0.438 | 0.418 | 0.046 | 3.787 |
| Sex= female | | 0.001 | 0.2364 | -0.462 | 0.464 | 0.000 | 1 | 0.997 | 1.001 | 0.630 | 1.591 |
| Sex=male | | 0^a^ |  |  |  |  |  |  | 1 |  |  |
| Income 0-5000₺ | | 0.293 | 0.3527 | -0.399 | 0.984 | 0.689 | 1 | 0.406 | 1.340 | 0.671 | 2.676 |
| Income 6-10000₺ | | 0.332 | 0.3331 | -0.320 | 0.985 | 0.996 | 1 | 0.318 | 1.394 | 0.726 | 2.679 |
| Income 11-15000₺ | | 0.311 | 0.3542 | -0.383 | 1.005 | 0.771 | 1 | 0.380 | 1.365 | 0.682 | 2.732 |
| Income 16-20000₺ | | -0.205 | 0.3998 | -0.988 | 0.579 | 0.263 | 1 | 0.608 | 0.815 | 0.372 | 1.784 |
| Income >20000₺ | | 0^a^ |  |  |  |  |  |  | 1 |  |  |
| Type of dystonia (BPS/HFS) | | 0.130 | 0.6120 | -1.070 | 1.329 | 0.045 | 1 | 0.832 | 1.139 | 0.343 | 3.779 |
| Type of dystonia (CD) | | 0.214 | 0.6161 | -0.993 | 1.422 | 0.121 | 1 | 0.728 | 1.239 | 0.370 | 4.146 |
| Type of dystonia (OMD) | | 1.113 | 0.7225 | -0.303 | 2.529 | 2.374 | 1 | 0.123 | 3.044 | 0.739 | 12.543 |
| Type of dystonia (SegD) | | 0.259 | 0.7281 | -1.168 | 1.686 | 0.127 | 1 | 0.722 | 1.296 | 0.311 | 5.399 |
| Type of dystonia (TSD) | | 0^a^ |  |  |  |  |  |  | 1 |  |  |
| Travel = yes | | 0.300 | 0.1909 | -0.074 | 0.674 | 2.476 | 1 | 0.116 | 1.350 | 0.929 | 1.963 |
| Travel = no | | 0^a^ |  |  |  |  |  |  | 1 |  |  |
| Private center = yes | | 0.857 | 0.1760 | 0.512 | 1.202 | 23.707 | 1 | **0.000** | 2.356 | 1.669 | 3.326 |
| Private center = no | | 0^a^ |  |  |  |  |  |  | 1 |  |  |
| Incorrect diagnosis= yes | | 0.746 | 0.2788 | 0.199 | 1.292 | 7.152 | 1 | **0.007** | 2.108 | 1.220 | 3.640 |
| Incorrect diagnosis= no | | 0^a^ |  |  |  |  |  |  | 1 |  |  |
| Occupation (housewife) | | -0.231 | 0.8705 | -1.937 | 1.475 | 0.070 | 1 | 0.791 | 0.794 | 0.144 | 4.373 |
| Occupation (employed) | | 0.123 | 0.8550 | -1.553 | 1.798 | 0.021 | 1 | 0.886 | 1.131 | 0.212 | 6.040 |
| Occupation (retired) | | -0.045 | 0.8855 | -1.781 | 1.690 | 0.003 | 1 | 0.959 | 0.956 | 0.168 | 5.421 |
| Occupation (unemployed) | | 0.606 | 1.0409 | -1.434 | 2.647 | 0.339 | 1 | 0.560 | 1.834 | 0.238 | 14.105 |
| Occupation (student) | | 0^a^ |  |  |  |  |  |  | 1 |  |  |
| Age | | -0.023 | 0.0073 | -0.037 | -0.009 | 10.183 | 1 | **0.001** | 0.977 | 0.963 | 0.991 |
| Education (years) | | -0.047 | 0.0225 | -0.091 | -0.003 | 4.327 | 1 | **0.038** | 0.954 | 0.913 | 0.997 |
| Symptom duration | | 0.020 | 0.0119 | -0.003 | 0.043 | 2.882 | 1 | 0.090 | 1.020 | 0.997 | 1.044 |
| Delay in diagnosis | | 0.120 | 0.0352 | 0.051 | 0.189 | 11.591 | 1 | **0.001** | 1.127 | 1.052 | 1.208 |
| (Scale) | | 1^b^ |  |  |  |  |  |  |  |  |  |
| Dependent Variable: Diagnostic burden. Predicted probability is for burden. | | | | | | | | | | | |

Supp-table-4: Parameter estimates for the logistic regression analysis to detect factors associated with application to private clinics for BoNT treatment.

|  | **B** | **S.E.** | **Wald** | **df** | **Sig.** | **Exp(B)** | **95% CI** | |
| --- | --- | --- | --- | --- | --- | --- | --- | --- |
| Age | 0.000 | 0.007 | 0.005 | 1 | 0.946 | 1.000 | 0.988 | 1.013 |
| Male sex | 0.012 | 0.191 | 0.004 | 1 | 0.949 | 1.012 | 0.696 | 1.472 |
| Education (years) | 0.004 | 0.022 | 0.038 | 1 | 0.845 | 1.004 | 0.963 | 1.048 |
| Income 0-5000₺ |  |  | 16.166 | 4 | 0.003 |  |  |  |
| Income 6-10000₺ | 0.305 | 0.229 | 1.775 | 1 | 0.183 | 1.357 | 0.866 | 2.124 |
| Income 11-15000₺ | 0.885 | 0.284 | 9.686 | 1 | 0.002 | 2.423 | 1.388 | 4.230 |
| Income 16-20000₺ | 0.988 | 0.352 | 7.874 | 1 | 0.005 | 2.687 | 1.347 | 5.359 |
| Income >20000₺ | 1.151 | 0.378 | 9.274 | 1 | 0.002 | 3.160 | 1.507 | 6.627 |
| Comes from the city center |  |  | 7.816 | 2 | 0.020 |  |  |  |
| City district | 0.167 | 0.253 | 0.436 | 1 | 0.509 | 1.181 | 0.720 | 1.938 |
| Another city | 0.562 | 0.201 | 7.788 | 1 | 0.005 | 1.753 | 1.182 | 2.601 |
| Type of dystonia (BPS/HFS) |  |  | 9.269 | 4 | 0.055 |  |  |  |
| Type of dystonia (CD) | 0.503 | 0.195 | 6.662 | 1 | 0.010 | 1.654 | 1.129 | 2.423 |
| Type of dystonia (OMD) | 0.059 | 0.391 | 0.023 | 1 | 0.880 | 1.061 | 0.493 | 2.283 |
| Type of dystonia (SegD) | 0.011 | 0.432 | 0.001 | 1 | 0.980 | 1.011 | 0.434 | 2.358 |
| Type of dystonia (TSD) | -0.751 | 0.816 | 0.847 | 1 | 0.357 | 0.472 | 0.095 | 2.337 |
| Not satisfied with BoNT treatment | 0.844 | 0.330 | 6.542 | 1 | 0.011 | 2.327 | 1.218 | 4.444 |
| Constant |  |  |  |  |  |  |  |  |
| Dependent Variable: Private center application for BoNT treatment. Predicted probability is for “Private center application= yes”. | | | | | | | | |

Supp-table-5: Parameter estimates for the logistic regression analysis to detect factors associated with satisfaction from BoNT treatment.

|  | **B** | **S.E.** | **Wald** | **df** | **Sig.** | **Exp(B)** | **95% CI** | |
| --- | --- | --- | --- | --- | --- | --- | --- | --- |
| Age | 0.001 | 0.012 | 0.012 | 1 | 0.914 | 1.001 | 0.978 | 1.025 |
| Male sex | -0.069 | 0.364 | 0.036 | 1 | 0.849 | 0.933 | 0.457 | 1.904 |
| Education (years) | -0.058 | 0.037 | 2.426 | 1 | 0.119 | 0.944 | 0.877 | 1.015 |
| Type of dystonia (BPS/HFS) |  |  | 10.804 | 4 | 0.029 |  |  |  |
| Type of dystonia (CD) | 0.376 | 0.394 | 0.912 | 1 | 0.340 | 1.456 | 0.673 | 3.150 |
| Type of dystonia (OMD) | 1.217 | 0.572 | 4.530 | 1 | **0.033** | 3.377 | 1.101 | 10.358 |
| Type of dystonia (SegD) | 1.365 | 0.640 | 4.558 | 1 | **0.033** | 3.917 | 1.118 | 13.721 |
| Type of dystonia (TSD) | 2.193 | 0.897 | 5.970 | 1 | **0.015** | 8.961 | 1.543 | 52.036 |
| Frequency of injections | -0.211 | 0.157 | 1.807 | 1 | 0.179 | 0.810 | 0.595 | 1.102 |
| Private center for BoNT | 0.817 | 0.330 | 6.114 | 1 | **0.013** | 2.263 | 1.185 | 4.324 |
| Alternative medicine | 0.650 | 0.443 | 2.152 | 1 | 0.142 | 1.916 | 0.804 | 4.568 |
| Constant |  |  |  |  |  |  |  |  |
| Dependent Variable: Satisfaction for BoNT treatment. Predicted probability is for unsatisfaction. | | | | | | | | |

Supp-table-6: Satisfaction from the BoNT and using alternative medicine in types of dystonia.

|  | BPS/HFS (n=347) | CD  (n=339) | OMD  (n=46) | SegD  (n=43) | TSD  (n=12) |
| --- | --- | --- | --- | --- | --- |
| Satisfied with BoNT treatment, % (n) | | | | | |
| Very satisfied | 55.9 (195) | 45.4 (154) | 47.8 (22) | 37.2 (16) | 50 (6) |
| Satisfied | 39.8 (139) | 48.4 (164) | 41.3 (19) | 51.2 (22) | 33.3 (4) |
| Not satisfied | 4.0 (14) | 4.1 (14) | 10.9 (5) | 11.6 (5) | 8.3 (1) |
| Not satisfied at all | 0 | 1.8 (6) | 0 | 0 | 8.3 (1) |
| Alternative medicine, % (n) | | | | | |
| Applied alternative medicine | 5.5 (19) | 16.9 (57) | 13.0 (6) | 11.9 (5) | 0 |
| Ozone therapy | 5.3 (1) | 10.7 (6) | 16.7 (1) | 0 | - |
| Leech therapy | 31.6 (6) | 21.4 (12) | 16.7 (1) | 20.0 (1) | - |
| Cupping | 52.6 (10) | 53.6 (30) | 33.3 (2) | 80.0 (4) | - |
| Acupuncture | 31.6 (6) | 35.7 (20) | 0 | 20.0 (1) | - |
| Fitotherapy | 5.3 (1) | 5.4 (3) | 16.7 (1) | 0 | - |
| Benefit from alternative medicine, % (n) | | | | | |
| Yes | 15.8 (3) | 1.8 (1) | 0 | 0 | - |
| Partially | 15.8 (3) | 17.5 (10) | 16.7 (1) | 40.0 (2) | - |
| No | 57.9 (11) | 63.2 (36) | 83.3 (5) | 60.0 (3) | - |
